# Investigation of *Trypanosoma evansi* infection in bullfighting cattle in Southern Thailand

**DOI:** 10.14202/vetworld.2020.1674-1678

**Published:** 2020-08-22

**Authors:** Ketsarin Kamyingkird, Piangjai Chalermwong, Vannarat Saechan, Domechai Kaewnoi, Marc Desquesnes, Ruttayaporn Ngasaman

**Affiliations:** 1 Department of Parasitology, Faculty of Veterinary Medicine, Kasetsart University, Ladyao, Chatuchak, Bangkok, Thailand; 2 Faculty of Veterinary Science, Prince of Songkla University, Chulabhorn karoonyaraksa Building, Hatyai, Songkhla, Thailand; 3 CIRAD, UMR InterTryp, Bangkok, Thailand; 4 InterTryp, Univ Montpellier, CIRAD, IRD, F 34398 Montpellier, France

**Keywords:** bullfighting cattle, seroprevalence, Thailand, *Trypanosoma evansi*

## Abstract

**Background and Aim:**

*Trypanosoma evansi* infection has been reported in Thai livestock such as beef and dairy cattle. However, there is little information on *T. evansi* infection in bullfighting cattle in Southern Thailand. The aim of this study was to investigate the infection of *T. evansi* in bullfighting cattle presented for health checks at the Animal Hospital, Faculty of Veterinary Science, Prince of Songkla University, Thailand.

**Materials and Methods:**

Blood and serum samples were collected from 177 bullfighting cattle from April 2016 to February 2017 after bullfighting matches. Animal inspected showed signs of fever, weight loss, or exercise intolerance. Investigation of *T. evansi* infection was tested using polymerase chain reaction (PCR) with TBR primers and using indirect enzyme-linked immunosorbent assay with *T. evansi* crude antigen.

**Results:**

The seroprevalence of *T. evansi* in bullfighting cattle was 22.60% (40/177). The PCR results detected no parasite DNA in this study. However, bullfighting cattle may serve as *T. evansi* reservoirs.

**Conclusion:**

Health checking procedures for *T. evansi* should be promoted for bullfighting events so that infected animals can be quarantined in the preparatory stages of such events.

## Introduction

*Trypanosoma evansi* is a haemoflagellate protozoan parasite. It is a causative agent of surra. It is widely prevalent in tropical and subtropical countries in North Africa, the Middle East, Asia, and South America [1,2]. It has a wide range of susceptible domestic and wild hosts such as horses, camels, buffaloes, domesticated cattle, and dogs [3]. *T. evansi* is transmitted mechanically by biting flies mostly belonging to the genera *Tabanus*, *Chrysops*, *Haematopota*, *Stomoxys*, *Haematobia*, and *Hippobosca* [4,5]. It causes clinical signs such as progressive emaciation, anemia, edema, pyrexia, reduced weight gains, and death in susceptible hosts. However, mild and subclinical infection in reservoir hosts is not readily apparent. In some countries, the incidence of surra increases significantly in the dry season when there are dense populations of biting flies [6]. In Thailand, *T. evansi* infections have been reported in cattle, water buffalo, elephants, horses, and dogs [7-9]. The overall prevalence of *T. evansi* infection in dairy cows was 8.1%, and herd prevalence was 19.2% in dairy cows Central Thailand [10]. The seroprevalence of *T. evansi* has been reported to be 50% in native cattle [11]. A mean seroprevalence of 12.2% was also reported in buffalo [9]. The infection has an economic impact on herds on both small-scale and large-scale farms [12]. Subclinical infection of *T. evansi* decreases the income of Thai farmers due to milk reduction [13].

Bullfighting is a non-lethal traditional sport between bulls and cows. It is also known as bull wrestling or cow fighting. This event is found in many countries, such as in some parts of Spain, Turkey, Croatia, Bosnia, Oman, the United Arab Emirates, India, Korea, Japan, China Vietnam, Lao, and Thailand [14-17]. In general, bulls are selected by age, horn length, horn size, and the number of fights [18]. Local cattle (*Bos indicus*) in Southern Thailand are raised as draft animals for agricultural activity, for meat consumption, and also for traditional bullfighting purposes [19]. The maximum weight in local cattle is in the range of 230-250 kg for females and 350-450 kg for males [19]. Male cattle aged 3-4 years are selected, trained, and prepared for bullfighting events [18]. Nowadays, bullfighting events are popular, with many people attending competitions held every ­weekend [20]. There are 28 legal and 32 illegal bullfighting arenas in Southern Thailand. Bulls are phenotypically selected for their large size and strength to act as fighting bulls [18]. During their training, moving, and gathering in the arena, bulls may be exposed to biting insects, which are potential carriers of *T. evansi*. The bulls are not given a health check, nor are blood samples taken and analyzed before they enter the arena. This may allow the transmission of surra among the beasts during training, gathering, and fighting in the bullfighting arena.

However, there is no information on *T. evansi* infection in bullfighting cattle in Southern Thailand. Therefore, the aim of this study was to investigate *T. evansi* infection in bullfighting cattle in Southern Thailand. The information will be useful for the establishment of the bullfighting cattle health-check regulations regarding movement, arranged bullfighting in the arena, and herding issues associated with bullfighting events.

## Materials and Methods

### Ethical approval

This study was approved by the Ethics Committee of Kasetsart University (Approval no. ACKU 01360).

### Animals and blood sample collection

Bullfighting cattle were sampled aged 3-5 years, presenting signs of fever, weight loss or weakness, and exercise intolerance. The blood samples of 177 bullfighting cattle were collected from April 2016 to February 2017 at the Prince of Songkla University Animal Hospital was included in this study. Ten milliliters of the blood sample was collected from the jugular vein of each animal and stored in ethylenediaminetetraacetic acid (EDTA) and dry tubes for polymerase chain reaction (PCR) and serological testing, respectively. The blood and serum samples were stored at −20°C until used for DNA extraction and indirect enzyme-linked immunosorbent assay (ELISA), respectively.

### DNA extraction and molecular diagnosis of *T. evansi*

DNA extractions were performed using commercial DNA extraction kits (Qiagen^®^, QIAamp^®^ DNA Blood Mini Kit, Maryland, USA), according to the manufacturer’s instructions. PCR was conducted using TBR1/2 primers to amplify a 164 bp product from a highly repeated sequence of mini-chromosome satellite DNA for the detection of *Trypanozoon* (Table-1), following a protocol previously described [21,22]. Briefly, 18 μL PCR mixtures were prepared using PCR buffer, 50 mM MgCl_2_, 10 mM dNTPs mixed, 10 μM of each primer, 0.5 Unit of taq DNA polymerase (Invitrogen™, Life Technologies, California, USA), and 2 μL of DNA sample. *Trypanosoma evansi* DNA was extracted from the blood of an experimentally infected mouse for the positive control, and distilled water was used as the negative control. Initial denaturation was 94°C 1 min followed by 30 cycles of denaturation at 94°C 30 s, annealing at 60°C 1 min, extension at 72°C 30 s, and an extra final extension at 72°C 2 min. PCR product was stained using non-toxic nucleic acid stains (Ultrapower^®^, BioTeke, Beijing, China) and migrated through 1.5% agarose gel (Biotech™, Bio Basic Canada Inc., Ontario, Canada) in a submarine electrophoresis system (Advance Mupid-exU TM, Takara Bio Inc., California, USA). PCR products were observed under a UV transilluminator (InGenius™, Syngene Bio Imaging^®^, Labnet International Inc., Woodbridge, USA).

**Table-1:** Primer and PCR conditions used in this study.

Primer name	Nucleotide sequence	Annealing temperature (°C)	DNA product
TBR1	5´GAATATTAAACAATGCGCAG 3´	60	164 bp
TBR2	5´CCATTTATTAGCTTTGTTGC 3´		

### Indirect ELISA for *T. evansi* antibody detection

Indirect ELISA using *T. evansi* crude antigen (TEC Thai isolate antigen) was conducted as previously described [6]. Briefly, *T. evansi* antigen was coated in a 96-well plate (Nunc^®^, Roskilde, Denmark) at 5 mg/mL in coating buffer and incubated at 37°C for 2 h Blocking buffer (7% skim milk) was used to reduce non-specific bindings. Sera samples were diluted 1:150 in blocking buffer and transferred into a 96-well plate as duplicates and incubated at 37°C under permanent shaking for 30 min. Secondary mouse anti-bovine IgG antibody was added (1:2,000 dilution) after a washing step using 0.01% tween in PBS 1× and incubated at 37°C with permanent shaking for 30 min. One hundred microliters of the ­complex substrate/chromogen 3,3′,5,5′-tetramethylbenzidine (TMB) (SureBlue™ TMB, KPL^®^, Maryland, USA) were added after a washing step and incubated at room temperature in a dark place for 30 min, and the optical density (OD) was read under an ELISA plate reader (Dynex Technologies^®^, VA, USA) at a wavelength of 620 nm. The relative percentage of positivity (RPP) was calculated as: RPP of a sample = (mean OD sample - mean OD of negative controls)/(mean OD of positive controls - mean OD of negative controls). A cutoff value of 20% was used as previously established for cattle in Thailand [8].

## Results

**Figure-1:**
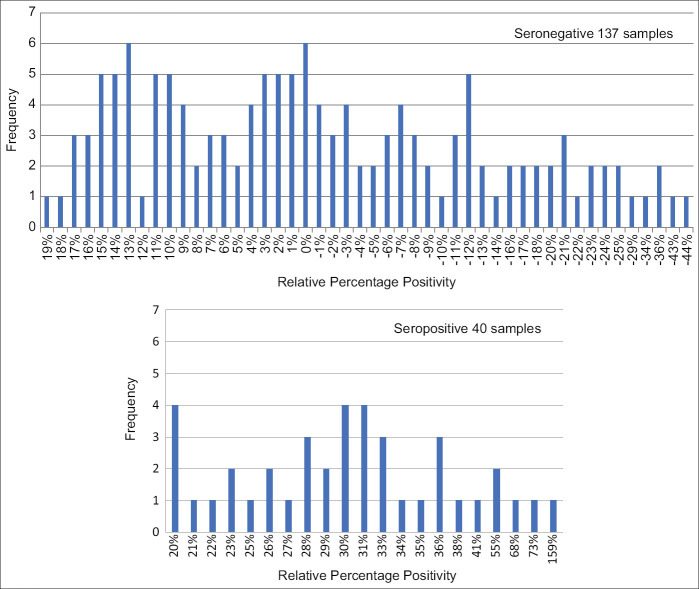
Relative percentage positivity levels of serum samples tested using indirect enzyme-linked immunosorbent assay (cutoff value at 20%).

Molecular detection of *T. evansi* infection using specific *Trypanozoon* satellite DNA showed that all 177 bullfighting cattle were negative. Indirect ELISA results showed that there were 40 (22.60%) seropositive bullfighting cattle for *T. evansi* infection. The seronegative analysis revealed that the RPP level was in the range of −44% to 19%, while the RPP level of seropositive samples was ranged from 20% to 156% (Figure-1). From those seropositive samples, 17 had exhibited a low RPP level (20% ≥ RPP < 30%), but 23 samples had a high RPP level (RPP ≥ 30%). However, the samples with low RPP levels were considered seropositive but require further laboratory confirmation. Our study revealed that bullfighting cattle may be exposed to *T. evansi* but not to an active infection. The contradiction between molecular and indirect ELISA results might have been due to the previous exposure to *T. evansi* but not having current *T. evansi* infection, since specific antibodies remain in animal blood circulation even after the parasite has been destroyed.

## Discussion

In Thailand, the seroprevalence of *T. evansi* infections has been reported in dairy cattle, beef cattle, buffaloes, and domestic elephants, with the prevalence ranging from 2.1% to 80% [7-9,23]. However, there have no report of *T. evansi* in bullfighting cattle. The current study reported for the first time on the seroprevalence of *T. evansi* infection in bullfighting cattle. In general, the sensitivity and specificity of indirect-ELISA for trypanosomes are higher than 90% [9]. Previously, indirect ELISA using crude *T. evansi* antigen was performed. In experimentally infected cattle, indirect ELISA showed satisfactory sensitivity and specificity [24]. Using the same indirect ELISA protocol, the seroprevalence levels of *T. evansi* infection in dairy cattle and buffaloes were 11.9% and 12.12%, respectively [8,9]. In comparison, seroprevalence in the current study was higher than in the previous studies, indicating that bullfighting cattle may be exposed to infection.

*T. evansi* infection in native cattle usually shows mild symptoms and occasionally may present with the nervous symptoms, including circling, excitation, jumping, aggressive behavior, recumbence, convulsion, and final death [25]. Clinical trypanosomosis, generally due to hemolysis from the oxidation of the erythrocytes, causes anemia [26]. Subclinical trypanosomosis is responsible for decreased milk yield in newly infected dairy cows [13]. Subclinical infections of *T. evansi* in bullfighting cattle may result in exercise intolerance, power reduction, or lack of strength for bullfighting. Lower fertility and higher mortality rates were also reported in areas of high *T. evansi* seroprevalence [27]. Young bulls may have lower growth rates. Moreover, the cumulative risk factors such as mixed-species rising of livestock, poor quarantine and hygiene practice, environmental factors, and high density and distribution of blood-sucking vectors may produce severe physiological and somatic stresses which lead to immunosuppression and a subsequent relapse in clinical signs.

In our study, bullfighting cattle presented with fever, weight loss, weakness, and exercise intolerance. Surprisingly, we did not detect active infection in any animals. The PCR results showed that the bullfighting cattle were not actively infected with *T. evansi*. However, a bull may serve as a reservoir for *T. evansi* as clinical signs are not usually detectable. Clinical signs indicating a health check are required, such as fever, weight loss, weakness, and exercise intolerance after the fighting might be due to another infectious disease, which needs further investigation. Among the seropositive bullfighting cattle, some could develop as reservoir hosts and become actively infected with *T. evansi*.

Bullfighting is a well-established cultural sport using locally bred cattle in some areas in Thailand [18]. In Southern Thailand, this sport is popular and is attractive for tourism and economic benefits [20]. For a bullfighting event, bulls are selected, trained, and matched before the competition [18]. The bulls are transported to and held at the arena before the fight. While animals generally undergo a physical inspection, there is no regulation requiring blood sampling before a fight [18]. In the arena, the transmission of *T. evansi* through biting insects may occur. This study showed that bullfighting cattle carried *T. evansi* antibodies and could develop as a reservoir host. To ^­^control *T. evansi* infection, our results suggest that quarantining due to *T. evansi* infection in bullfighting cattle should be performed before any affected animals are allowed to be transported to or trained or matched at a bullfighting event.

## Conclusion

Bullfighting cattle are exposed to *T. evansi*. To control and prevent *T. evansi* infection in bullfighting cattle, this study suggested that blood sampling and analysis should be regulated, especially for the detection of *T. evansi*, before transporting, herding animals together or matching for bullfighting. Due to the limitations of this study, further work is needed to determine the extent of *T. evansi* infection in the greater population of bullfighting cattle.

## Authors’ Contributions

KK carried out laboratory diagnosis, data analysis, manuscript preparation, revision, and submission of the manuscript. PC carried out a laboratory diagnosis. VS and DK did the sample collection. MD provided diagnostic reagents and supervised and revised the manuscript. RN designed the study, analyzed data, revised the manuscript, and contributed to the study. All authors have read and approved the final manuscript.

## Competing Interests

The authors declare that they have no competing interests.

## Publisher’s Note

Veterinary World remains neutral with regard to jurisdictional claims in published institutional affiliation.
